# Regulatory Lymphoid and Myeloid Cells Determine the Cardiac Immunopathogenesis of *Trypanosoma cruzi* Infection

**DOI:** 10.3389/fmicb.2018.00351

**Published:** 2018-03-01

**Authors:** Manuel Fresno, Núria Gironès

**Affiliations:** ^1^Centro de Biología Molecular Severo Ochoa (CSIC), Madrid, Spain; ^2^Instituto de Investigación Sanitaria del Hospital Universitario de La Princesa, Madrid, Spain

**Keywords:** Chagas disease, myocarditis, immunoegulation, regulatory T cells, Th1 cells, Th17 cells, *Trypanosoma cruzi*, MDSCs

## Abstract

Chagas disease is a multisystemic disorder caused by the protozoan parasite *Trypanosoma cruzi*, which affects ~8 million people in Latin America, killing 7,000 people annually. Chagas disease is one of the main causes of death in the endemic area and the leading cause of infectious myocarditis in the world. *T. cruzi* infection induces two phases, acute and chronic, where the infection is initially asymptomatic and the majority of patients will remain clinically indeterminate for life. However, over a period of 10–30 years, ~30% of infected individuals will develop irreversible, potentially fatal cardiac syndromes (chronic chagasic cardiomyopathy [CCC]), and/or dilatation of the gastro-intestinal tract (megacolon or megaesophagus). Myocarditis is the most serious and frequent manifestation of chronic Chagas heart disease and appears in about 30% of infected individuals several years after infection occurs. Myocarditis is characterized by a mononuclear cell infiltrate that includes different types of myeloid and lymphoid cells and it can occur also in the acute phase. *T. cruzi* infects and replicates in macrophages and cardiomyocytes as well as in other nucleated cells. The pathogenesis of the chronic phase is thought to be dependent on an immune-inflammatory reaction to a low-grade replicative infection. It is known that cytokines produced by type 1 helper CD4+ T cells are able to control infection. However, the role that infiltrating lymphoid and myeloid cells may play in experimental and natural Chagas disease pathogenesis has not been completely elucidated, and several reports indicate that it depends on the mouse genetic background and parasite strain and/or inoculum. Here, we review the role that T cell CD4+ subsets, myeloid subclasses including myeloid-derived suppressor cells may play in the immunopathogenesis of Chagas disease with special focus on myocarditis, by comparing results obtained with different experimental animal models.

## Introduction

*Trypanosoma cruzi* life cycle involves stages in invertebrate and vertebrate hosts including domestic and wild species. Clinically *T. cruzi* infection can be divided in two phases, acute and chronic. In the acute phase, after infection, a local inflammatory lesion can appear at the site of the bite. Death occurs occasionally in the acute phase (<5–10% of symptomatic cases) as a result of severe myocarditis or meningoencephalitis, or both. But frequently this phase shows very mild and unspecific symptoms and the infection can pass easily unnoticed. About 60–70% of the infected people will never develop clinically apparent disease being asymptomatic (undetermined). The remaining (around 30%) of patients develop the most severe chronic pathology of the disease that appears between one and three decades after primary infection with *T. cruzi* (Rassi et al., 2010; Perez-Molina and Molina, 2017; Telleria and Tibayrenc, 2017).

*T. cruzi* is a heterogeneous species with a high genetic variability being classified into six Discrete Typing Units (DTUs), also named TcI to TcVI, which have been associated with geographical distribution, transmission cycle (domestic and sylvatic) but it has also been proposed that different DTUs are associated with diverse clinical manifestations (Zingales et al., 2009, 2012).

*T. cruzi* infection elicits a complex immune response. Different cell types can recognize *T. cruzi* and react on different ways for controlling infection. Detection and direct destruction of parasites by phagocytes, like macrophages and dendritic cells, which are activated to become APCs and initiate the adaptive immune response is fundamental. Also non-hematopoietic cells, as primary targets of invasion, can sense infection and can contribute to control the infection (Telleria and Tibayrenc, 2017).

Upon *T. cruzi* infection macrophages secrete interleukin (IL)-12, which activates natural killer (NK) cells to produce interferon (IFN)-γ (Aliberti et al., 1996; Antúnez and Cardoni, 2000), which plays a crucial role in activation of macrophages acting synergistically with TNF-α to express inducible nitric oxide synthase (iNOS) (Muñoz-Fernández et al., 1992) and Cyclooxygenase-2 (COX-2) (Guerrero et al., 2015).

## CD4+ T cell subsets

T cells originate from bone marrow T cell precursors that enter circulation and reach the thymus, where they differentiate into different types as CD4+ helper (Th) and regulatory (Treg), CD8+ cytotoxic (CTLs), and natural killer (NKT) T cells that colonize secondary lymphoid organs and tissues. There are several studies about the role of CD4+ and CD8+ cells in *T. cruzi* infection (reviewed in Cardillo et al., 2015). In general those studies have provided evidence on the protective role of Th1, especially on the outcome of acute infection *in vivo* (Holscher et al., 1998). On the other hand, Th2/antibody specific response seems to play a minor role on protection (Abrahamsohn et al., 2000). Although it was initially published that perforin/granzyme mediated killing was not necessary for resistance to infection (Kumar and Tarleton, 1998), subsequent studies reported that CTLs are important to control intracellular infection through perforin/granzyme mediated killing of infected cells and/or FAS-mediated apoptosis (Müller et al., 2003; Martin and Tarleton, 2004; Silverio et al., 2010). We will focus this review on inflammatory and regulatory CD4+T cell subset studies (summarized in Table 1).

**Table 1:** Lymphoid immune responses to *T. cruzi* infection in different mouse models.

**Mouse**	**Parasite**	**Organ**	**Approach**	**Th1/Th2**	**Treg**	**Th17**	**References**
BALB/c	Y	Heart		Low/Exacerbation			Cuervo et al., 2008
BALB/c	Y	Heart		Low/Exacerbation		High/Exacerbation	Sanoja et al., 2013
BALB/c	Tulahuén	Heart Spleen	Neutrophil depletion	Low/Exacerbation			Chen et al., 2001
BALB/c	Y	Heart	Anti-CD25		Limited role		Mariano et al., 2008
BALB/c	H8 Yucatan	Spleen	rSSP4 immunization		rSSP4 specific/Exacerbation		Flores-García et al., 2013
BALB/c	Y	Spleen Heart	Anti-IL-17			High/Protective	da Matta Guedes et al., 2010
BALB/c TS-CD4-Tg	Tulahuén	Spleen	Adoptive transfer of TS specific T cells into RAG KO	High/ Less protection		High/More protection	Cai et al., 2016
C57BL/6	Y	Heart		High/Protection			Cuervo et al., 2008
C57BL/6	Y	Heart		High/Protection	High/Protection	High/Protection	Sanoja et al., 2013
C57BL/6	Tulahuén	Heart Spleen	Neutrophil depletion	High/Protection			Chen et al., 2001
C57BL/6	Y	Spleen	Osteopontin antibody	High/Protection		High/Protection	Santamaría and Corral, 2013
C57BL/6	Tulahuén	Spleen	Anti-IL-10	NA	NA	NA	Silva et al., 1992
C57BL/6	Colombiana	Heart	IL-10 KO	NA	NA	NA	Roffê et al., 2012
C57BL/6	Tulahuén	Thymus periphery			Low/Exacerbation		González et al., 2016
C57BL/6	Colombian	Heart	G-CSF treatment		High/Protection		Vasconcelos et al., 2013
C57BL/6	Y	Heart	SOCS2 KO		Low/Protection pathology		Esper et al., 2012
C57BL/6	Tulahuén	Liver Heart Spleen	Ebi3 KO (classical Treg cells)	High/Protection		High/Protection	Böhme et al., 2016
C57BL/6	Y	Heart	Ebi3 KO (Tr1 cells)		High/Protection		Medina et al., 2017
C57BL/6	Tulahuén	Spleen		High/Exacerbation	Low/Exacerbation		González et al., 2015
C57BL/6	Tulahuén	Spleen	IL-6 KO	NA	NA	NA	Gao and Pereira, 2002
C57BL/6	Tulahuén	Spleen	BATF2 KO			High/Protection	Kitada et al., 2017
C57BL/6	Tulahuén	Liver	IL-17A KO			High/Protection	Miyazaki et al., 2010
C57BL/6	Tulahuén	Spleen LN Liver	IL-17RA KO			High/Protection	Tosello Boari et al., 2012
C3H/HeN	RA		Tc52/immunized	High/protection		High/protection	Matos et al., 2017
C57BL/6 B6.SJL C3H/HeSnJ	Tulahuén	Muscle Brain Gut	Anti-CD25		Limited role high/protect		Kotner and Tarleton, 2007
C57BL/6 C3H/HeJ BALB/c	Colombian Y	Heart	Anti-CD25		Limited role		Sales et al., 2008
A/J	Brazil	Heart	Anti-CD25		High/Exacerbation		Bonney et al., 2015
Swiss Webster	Colombian		Anti-CD25		High/Exacerbation		Nihei et al., 2014
C3H	Sylvio X10/4 clone	Heart	G-CSF Benznidazol treatment	Low/Pathology	Medium/Pathology	High/Pathology	González et al., 2013

*Mouse and parasite strain used; organ studied; approach used: infection of deficient mouse strain or knockout (KO), treatment, immunization, cell depletion, or adoptive transfer; Th1/Th2 balance Treg and Th17 responses (high medium or low)/effect (Protection, exacerbation or pathology) and references are indicated*.

### Th1/Th2 cells

It is known that type 1 CD4+T cell (Th1) response mediated by pro-inflammatory cytokines as IFN-γ, Tumor necrosis factor (TNF) and IL-1β, is protective against *T. cruzi* infection, in macrophages *in vitro* (Gazzinelli et al., 1992; Muñoz-Fernández et al., 1992) and *in vivo* using IFN-γ receptor KO mice (Holscher et al., 1998; Aliberti et al., 2001). It has been found that IL-10 is required to prevent an excessive pro-inflammatory response during *T. cruzi* infection, and something similar occurs with Transforming growth factor (TGF)-β (Silva et al., 1991) although this cytokine might have other functions besides immune regulation (Ming et al., 1995; Hall and Pereira, 2000). It has been proposed that IL-4 also downregulates IFN-γ and inflammation when cooperating with IL-10 (Abrahamsohn et al., 2000), but some other reports showed that this cytokine has similar effects to those of IFN-γ as trypanocidal activity (Wirth et al., 1989; Golden and Tarleton, 1991). Although the role of IL-13 is not clear yet, it has been documented that this cytokine might also be involved in the regulation of IFN-γ release (Antúnez and Cardoni, 2001).

However, the parasite is able to trigger both responses in different magnitude depending on the mouse genetic background, parasite strain, and inoculum. By comparing susceptible and non-susceptible mice (BALB/c and C57BL/6, respectively) infected with the highly virulent Y strain of the parasite we evidenced that protection depends on the Th1/Th2 balance (Cuervo et al., 2008; Sanoja et al., 2013). Thus, production of cytokines by T cells infiltrating the heart at the peak of parasite infection, presented higher Th1/Th2 cytokine balance in mice non-susceptible to infection than in susceptible mice.

In agreement, other groups, using different approaches, observed for instance that low Th1/Th2 balance after neutrophil depletion in BALB/c infected with Tulahuén strain exacerbated the infection, indicating that a high Th1/Th2 balance is protective against the infection, but the opposite effect was found when infecting C57BL/6 with the same parasite strain (Chen et al., 2001). In agreement, co-cultures of *T. cruzi* infected macrophages with live neutrophils isolated from BALB/c and C57BL/6 resulted also in increased and decreased parasite replication, respectively (Luna-Gomes et al., 2014), indicating the importance of the mouse genetic background in *T. cruzi* infection. In addition, anti osteopontin antibody treatment in C57BL/6 infected with the Y strain (Santamaría and Corral, 2013) and *T. cruzi* TC52 antigen immunized C3H/HeN mice infected with the RA strain (Matos et al., 2017), induced a high Th1 response that protected mice from infection. However, it is important to point out that the development of severe CCC in humans is also thought to be due to a Th1-specific immune response (Gomes et al., 2003), thus some regulation seems to be needed to avoid disease progression. In this direction, studies in C57BL/6 mice showed a regulatory role of IL-10 produced by T cells in spleen of mice infected with the Tulahuén strain (Silva et al., 1992) and a protective role in mice infected with the Colombiana strain (Roffê et al., 2012).

### Treg/Th17 cells

As mentioned above, Treg cells may help to control T cell responses during infection. Natural (n)Treg are able to maintain self-tolerance in the thymus (Sakaguchi et al., 1995), but (i)Treg cells can also be induced in response to infection by microorganisms, and are characterized by the expression of CD25, the transcription factor forkhead box P3 (FOXP3), and production of anti-inflammatory IL-10 and TGF-β (Jäger and Kuchroo, 2010). On the other hand T helper (Th17) cells, characterized by pro-inflammatory IL-17 and IL-23 production, are associated with autoimmune diseases (Wynn, 2005). Reciprocal developmental pathways have been described for the generation of both Treg and Th17 cells, Treg require IL-2, and TGF-β for differentiation while Th17 require TGF-β and IL-6 (Bettelli et al., 2006). It was later described that Treg cells are induced as well by Epstein-Barr virus-induced gene 3 (Ebi3)/IL-35 heterodimers (Collison et al., 2010).

The role of Treg and Th17 cells in *T. cruzi* infection is not completely understood. It is worth mentioning that most studies have been done using very frequently the Tulahuén and Y strains in different mouse genetic background combinations (Table 1). Initial studies on Treg depletion with anti-CD25 antibodies in acute and chronic mouse experimental models involving C57BL/6-Tulahuén strain (Kotner and Tarleton, 2007; Sales et al., 2008) or BALB/c-Y strain (Mariano et al., 2008), suggested a limited role for Treg cells in the control of *T. cruzi* infection. Moreover, studies in A/J mice infected with the Brazil strain, and SwissWebster mice infected with the Colombian strain, suggested that high Treg levels produce exacerbation of the infection (Nihei et al., 2014; Bonney et al., 2015), respectively. In contrast, we have observed that relative expansion of Treg cells might confer protection to infection in a non-susceptible combination (C57BL/6 mice infected with the Y strain) that presented as well a high Th1/Th2 balance (Sanoja et al., 2013). In the same direction, the loss of thymic Treg cells during the infection in C57BL/6 mice with the Tulahuén strain compromise the peripheral pool that is related with immune dysregulation (González et al., 2016). In agreement with these observations it was described that treatment of C57BL/6 mice with G-CSF incremented Treg levels and conferred increased protection to infection with the Colombian strain (Vasconcelos et al., 2013). However, other reports showed that immunization with rSSP4, a parasite antigen, increased rSSP4 specific Treg and produced exacerbation of the infection with H8 Yucatan strain in BALB/c mice (Flores-García et al., 2013). In addition, studies using Suppressor of cytokine signaling 2 (SOCS2) deficient mice (C57BL/6 background) showed that low Treg levels protected from infection with the Y strain, while increased immunopathology (Esper et al., 2012). By infecting Ebi3 deficient mice (C57BL/6 background) with the Tulahuén strain it was shown that Ebi3 suppressed Th1, Th2, and Th17 responses during *T. cruzi* infection which protected mice by interfering with alternative macrophage activation (Böhme et al., 2016).

On the other hand, IL-27 produced by myeloid cells, is able to induce a different type of regulatory T cells that produce IL-10 (named Tr1 cells), in combination with Ebi3. It was recently described using Ebi3 deficient mice (C57BL/6 background) infected with the Y strain, that Ebi3 modulates IFN-γ mediated myocarditis, through IL-10, likely produced by Tr1 cells rather than classical Treg cells. These results in mice were in agreement with the presence of EBI3 polymorphisms in chagasic patients suffering severe cardiomyopathy (Medina et al., 2017).

Notably, others reported the existence of FOXP3+ cells that acquired a Th1-like phenotype in C57BL/6 mice results in exacerbation of infection with the Tulahuén strain (González et al., 2015) highlighting the importance of a regulated immune response.

Although several studies on regulatory T cells have been performed in animal models, there are also reports of the associations of those cells with cardiac dysfunction in chagasic patients that point out Treg cells as a potential therapeutic target (Mengel et al., 2016). We will briefly comment here some evidences about the importance of Treg cells in the chronic pathology. Thus, studies in groups of chagasic patients in different phases of the disease showed that Treg cells might play a role in the immune response against *T. cruzi* infection although with distinct effects in undetermined and cardiac patients (Araujo et al., 2007). In the same direction it was shown that individuals in the undetermined form of the disease have a higher frequency of Treg cells, suggesting that an expansion of those cells could be beneficial, possibly by limiting strong cytotoxic activity and tissue damage (de Araújo et al., 2011). Moreover, it was found that an immunological imbalance might be the cause of a deficient suppressor activity of regulatory T cells that controls myocardial inflammation (Guedes et al., 2012). Interestingly, studies performed with explants from patients with advanced chronic Chagas disease submitted to heart transplantation showed a skewed Th1/T cytotoxic profile whereas Treg cells were scarce and located only in areas of severe myocarditis (Argüello et al., 2014).

Our results on CD4+ T cells infiltrating heart tissue in mice (Sanoja et al., 2013) are in agreement with the data obtained from hearts of chagasic patients. Together, those studies indicate that a Th1 response seems necessary to clear the infection in the heart but if this response is unchecked by infiltrating Treg cells, may lead to excessive inflammation and CCC.

On the other hand, IL-17 producing Th17 cells have been shown to play a protective role against parasite-induced myocarditis in BALB/c mice infected with 100 blood trypomastigotes (low inoculum) of the Y strain per mice (susceptible model), by inhibiting Th1 differentiation during the acute phase of infection (da Matta Guedes et al., 2010). However, we found that in susceptible BALB/c mice high Th17 levels correlated with protection only with low parasite inocula (50 blood trypomastigotes of the Y strain per mice), but with high inocula (2000 blood trypomastigotes per mice) high Th17 levels were associated with high heart parasite burden, low heart CD4+ T cell infiltration and high mortality (Sanoja et al., 2013). However, previous reports showed that IL-6 was required for resistance of C57BL/6 mice infected with the Tulahuén strain (Gao and Pereira, 2002).

Recently, several reports using mice with C57BL/6 background infected with the Tulahuén strain showed that Th17 high levels conferred protection against infection using Il-23 inhibitory BATF2 (Kitada et al., 2017), regulatory EBI3 (Böhme et al., 2016), IL-17A (Miyazaki et al., 2010) and IL-17RA (Tosello Boari et al., 2012) deficient mice. On the other hand, immunization of C3H/HeN mice with TC52, a *T. cruzi* protein with glutathione transferase activity and a vaccine candidate, conferred Th17 specific protection against infection with the RA strain (Matos et al., 2017). Similarly, adoptive transfer of BALB/c parasite transialidase (TS) specific Th17 cells into RAG mice (lacking B and T cells) conferred higher protection than TS specific Th1 cells against infection with the Tulahuén strain (Cai et al., 2016).

Thus, different mechanisms seem to mediate protection depending on the mouse model and the *T. cruzi* strain and inoculum.

## Myeloid subsets

It was previously thought that macrophages originated from bone marrow monocytes, which in normal conditions differentiate in different types of tissue macrophages or become activated in inflammatory processes. More recently, it was described that resident tissue macrophages can have embryonic origin. In this direction, it has been recently proposed a new nomenclature for macrophages and dendritic cells based on ontology (Guilliams et al., 2014). However, we are going to focus on the role of macrophages, dendritic cells, and myeloid-derived suppressor cells (MDSCs) that can be of different origin but share localization and present similar phenotypes during *T. cruzi* infection (summarized in Table 2). It is worth mentioning the importance of myeloid cells as “Trojan horses” disseminating the parasite throughout different organs. This has been evidenced in Signaling Lymphocytic Activation Molecule family member 1 (SLAMF1) KO mice (BALB/c background) infected with the Y strain, in which myeloid cells are refractory to infection and all SLAMF1 KO mice survive while all BALB/c die from infection, suggesting a great contribution of myeloid cells in disseminating infection (Calderón et al., 2012). More recently, studies using *in vivo* imaging showed that bioluminiscent parasites are detected in different anatomical locations during infection in the absence of locally persistent infection, most likely inside infected myeloid cells, which is also in agreement with the “Trojan horse” hypothesis (Lewis et al., 2014).

**Table 2:** Myeloid immune responses to *T. cruzi* infection in mouse models.

**Mouse**	**Parasite**	**Organ**	**Approach**	**M1/M2**	**MDSCs**	**References**
C57BL/6	Tulahuén	Liver Heart Spleen	Ebi3 KO	Low/Exacerbation		Böhme et al., 2016
BALB/c	G	Spleen	IFNg KO iNOS KO iNOS inhibitor		High/Exacerbation	Goñi et al., 2002
BALB/c	Tulahuén	Heart	CD73 inhibitor	High/Protection		Ponce et al., 2016
B6.129S	Tulahuén	Fat		Low/Exacerbation		Cabalén et al., 2016
BALB/c	Dm28	PECs	CD8+ apoptotic cells	Low/Exacerbation		Cabral-Piccin et al., 2016
B6.129S	Tulahuén	Heart	IL-6 KO	Low/Exacerbation		Sanmarco et al., 2017
BALB/c	Y	Heart			High/Exacerbation	Cuervo et al., 2011
BALB/c C57BL/6	Tulahuén	Spleen Liver	MDSCs inhibitor 5FU		High/Protection	Arocena et al., 2014

*Mouse and parasite strain used; organ studied; approach used: infection of deficient mouse strain or knockout (KO), treatment, immunization, cell depletion, or adoptive transfer; M1/M2 balance and MDSCs responses/effect and references are indicated. Peritoneal macrophage (PECs)*.

### M1/M2 macrophages

Mirroring T cell behavior, monocytes can be activated by Th1 cytokines that convert them into classically activated macrophages (also named CAM or M1) that kill the parasite, or by Th2 cytokines that convert them into alternatively activated macrophages (also named AAM or M2) that promote proliferation (Munder et al., 1999).

As mentioned, in most cases resistance to the infection in the acute phase is associated with Th1 cells through IFN-γ production, which activates JAK/STAT pathway leading to STAT1α translocation and the transcription of a stable iNOS mRNA species in M1 macrophages. In turn, increased iNOS, which metabolizes L-arginine and produces NO, controls parasite replication (Muñoz-Fernández et al., 1992; Bergeron and Olivier, 2006). In addition, during acute infection there is suppression of T cell proliferation that is partially suppressed by NO (Goñi et al., 2002).

On the other hand, M2 macrophages, induced by Th2 and regulatory cytokines, have been implicated in parasite growth (Vincendeau et al., 2003). They express Arginase 1 that metabolizes L-arginine to produce ornithine that in turn is metabolized by ornithine decarboxylase (ODC) to produce polyamines needed for growth of all eukaryotic cells. In this regard, expression of arginase 1, a M2 marker (Ghassabeh et al., 2006), was found to be upregulated in macrophages infected with *T. cruzi* and associated to parasite survival (Stempin et al., 2004). Studies using EBI3 KO mice (C57BL/6 background), which showed decreased levels of Tregs after infection with the Tulahuén strain, also showed increased expression of arginase 1, which could indicate an exacerbated M2 polarization that was associated with susceptibility to infection (Böhme et al., 2016). Also, treatment of mice with anti-CD73, an enzyme involved in ATP generation, resulted in polarization toward M1 phenotype and protection of BALB/c mice against the Tulahuén strain (Ponce et al., 2016), and infection of B6.129S mice potentiated a M2 polarization in adipose tissue in a diet-induced obesity mouse model (Cabalén et al., 2016). Also apoptotic CD8+ T cells increased M2 differentiation contributing to parasite persistence in peritoneal macrophages from BALB/c mice infected with Dm28 strain (Cabral-Piccin et al., 2016). Finally, it has been described that IL-6 promotes M2 macrophage polarization that infiltrates heart tissue, downregulating NO production and reducing cardiac damage induced by infection with the Tulahuén strain in mice of mixed background (B6.129S) (Sanmarco et al., 2017).

### MDSCs and extracellular L-arginine

Besides M1 and M2 macrophages, during inflammation, trauma, cancer, and also infection, there is an interference of myeloid cell differentiation that induces the expansion and accumulation of immature myeloid cells (F4/80-), which are known as myeloid-derived suppressor cells (MDSCs) (Gallina et al., 2006; Gabrilovich and Nagaraj, 2009). While M2 macrophages are thought to express arginase 1 but no iNOS, MDSCs can express both enzymes (Gabrilovich and Nagaraj, 2009). MDSCs have been extensively studied in cancer biology, and they can eventually convert into Tumor associated macrophages (TAMs) that express the maturation marker F4/80 (Bronte et al., 2016). In addition, neutrophils can also convert into Tumor associated neutrophils (TANs) with tumor suppressor (N1) or pro-tumorigenic (N2) activity with parallelism with Th1/M1 and Th2/M2 responses (Bronte et al., 2016).

However, other authors defined CD11b+Gr-1(Ly6G/Ly6C)+ cell subsets as MDSCs, depending on their precursor origins: macrophages, granulocytes, and dendritic cells (DCs) (Geissmann et al., 2010), making classification even more complex. TipDCs, which are DCs that express iNOS and TNF and tumor tolerogenic DCs express arginase 1 have also been described (Rodriguez et al., 2017).

Arginase 1 and iNOS, either separately or in combination, can inhibit T-cell responses. L-Arginine is required for T cell proliferation and the threshold of L-arginine concentration in mammalian plasma that permits fully functional T cell proliferation is in the order of 100 μM (Choi et al., 2009). Moreover, combined activity of arginase 1 and iNOS enzymes has been shown to be important in the suppressive activity of mouse MDSCs in tumors (Bronte et al., 2003), but there are also several evidences of the role of MDSCs, iNOS, and L-arginine depletion in infectious diseases as chronic infections with helminthes (Brys et al., 2005). L-arginine is considered semi-essential in mammals as an extra contribution is required in the diet in stressed conditions such as pregnancy, trauma, or infection, in which the requirements of the amino acid exceeds the production capacity of the organism (Bronte and Zanovello, 2005).

MDSCs have been characterized by the expression of Ly6C and Ly6G surface antigens, that allow to classify them as monocytic CD11b+Ly6C+Ly6G- (M-MDSCs) or polymorphonuclear CD11b+Ly6C-Ly6G+ (PMN-MDSCs) cell subsets (being the last currently indistinguishable from TANs). They are further characterized by their capacity to suppress T cell proliferation. However, it has been recently proposed that subsets that do not suppress proliferation should be described as MDSC-like cells (MDSC-LC) (Bronte et al., 2016).

We have shown that acute *T. cruzi* infection induces alterations the appearance of CD11b+Ly6C+Ly6G- M-MDSC in spleen and heart (Goñi et al., 2002; Cuervo et al., 2008, 2011) of BALB/c mice infected with a the Y strain. Similar cells were found in spleen and liver of C57BL/6 mice infected with the Tulahuén strain (Arocena et al., 2014). In addition, these M-MDSCs cells express COX-2 and produce PGE_2_ that contribute significantly to heart leukocyte infiltration and to the release of chemokines and inflammatory cytokines in the heart of *T. cruzi* infected mice, being somehow detrimental for the host (Guerrero et al., 2015).

In the heart, we found leukocyte infiltration characterized by the presence of PMN-MDSCs that via S100A8/A9 may recruit M-MDSCs, expressing Arg-1 and iNOS, which suppress T cell proliferation (Cuervo et al., 2011). M-MDSCs expansion is linked to local and systemic extracellular L-arginine depletion, likely involved in immunosuppression. It is worth mentioning that a decrease in L-arginine causes down regulation iNOS expression (Konig et al., 2009) and reduces NO production by substrate competition. Notably, L-arginine administration to infected mice incremented plasma L-arginine and NO levels, significantly decreased parasitemia, heart parasite burden and clinical score, while increasing mice survival and cardiac performance (Carbajosa et al., 2018). In this direction, L-arginine treatment has been also shown to be beneficial in preventing *Trypanosoma cruzi* vertical transmission in rats (da Costa et al., 2014) and in patients affected by Dilated Cardiomyopathy, which clinically is the most similar to Chagas cardiomyopathy (Kralova et al., 2015).

In addition, inflammatory monocyte-derived dendritic cells (moDCs) have been recently found in skin after *T. cruzi* intradermal inoculation, which could be related with MDSCs (Poncini and Gonzalez-Cappa, 2017), showing the importance of these cells in the natural transmission of the parasite.

Moreover, after a global metabolomic analysis we found elevated levels of asymmetric+symmetric dimethyl-arginine (ADMA+SDMA) after infection in heart and plasma (Gironès et al., 2014) and confirmed elevated levels of ADMA in plasma (Carbajosa et al., 2018). ADMA is a product nuclear proteolysis by arginine methyltransferases (Teerlink, 2005), and is an endogenous inhibitor of iNOS that in combination with L-arginine regulates NOS activity (Blackwell, 2010). We have described that during *T. cruzi* infection there is L-arginine depletion and increased levels of ADMA. Thus, despite iNOS expression by MDSCs and M1 macrophages is high, its activity could be inhibited by the combination of lack of L-arginine as substrate and high levels of ADMA (Carbajosa et al., 2018). Interestingly, the L-arginine/ADMA ratio is a predictor of NO bioavailability and mortality in Dilated Cardiomyopathy (Anderssohn et al., 2012), a disease with some similarities with Chagasic Cardiomyotpathy, suggesting a possible relation with pathology.

Therefore, supplementation with L-arginine in the diet is beneficial for the infected mice in the acute phase of infection and, although further experiments are needed to go into the clinic. L-arginine can eventually have beneficial effects in patients, alone or in combination with antiparasitic drugs.

## Conclusion and perspectives

There are several aspects to be taken into account in the regulation of Cardiac Chagas immunopathogenesis. First, the dynamics of different subsets of immune cells is mutually dependent and interconnected. Parasite molecules that might trigger different immune responses depending on the parasite genotype because of the expression of virulence factors that facilitate infection of particular cell types and tissues. Because of being eukaryotic cells parasite possess many common pathways with the host and might alter several host pathways. In addition, most of the studies that describe immune lymphoid and myeloid cell subsets, have been done in inflammatory models in the absence of infection, and it is often difficult to fit the ones observed during real infections with those classifications.

In all studies in mice, it is important to note that parasite strain and mouse genetic background, as well as the amount of parasites, greatly determine the results of infection and also affects the presence or absence of those regulatory cells (Sanoja et al., 2013). Thus, parasite genome and mouse genetic background greatly determine the outcome of the infection, and this might explain the apparent discrepancies observed in the literature among different groups.

Nevertheless, in all instances immune regulation seems to be the key to fight infection, since a strong protective Th1 response can cause organ damage if not regulated. A similar statement can be formulated for Th17 response. In addition, resolution of inflammation by myeloid cells also may play a role. Ponce et al. described an initial infiltration of M1 macrophages into the heart, followed by expansion of M2 to heal the organ (Sanmarco et al., 2017). Likely, there is overlap between M1/M2 and MDSCs because studies were done analyzing only a few markers that are common to both cells types. An important point to take into account when comparing all those studies is the definition of the different myeloid cell subsets infiltrating the heart, which are based on cell surface marker expression. Thus, the main difference between mature M1 macrophages and MDSCs is the expression of F4/80 (marker of mature macrophages). However, in cancer models F4/80- MDSCs give rise later to tumor associated macrophages (TAMs) that express F4/80 (Bronte et al., 2016), thus in *T. cruzi* infection the possibility that MDSCs end up expressing this maturation marker cannot be discarded.

Most studies on *T. cruzi* infection were planed according current classifications based on markers which are mutually exclusive (i.e., T-Bet for Th1 cells, FOXP3 for Tregs, or RORγT for Th17 cells), however infection might trigger different types of cells, for instance, as mentioned, FOXP3+ T cells with a Th-1-like phenotype (González et al., 2015). In the other hand, different myeloid cells share common myeloid markers as CD11c, CD11b, and F4/80 (Guilliams et al., 2014). Thus, future work should take different subsets and markers into account in order to perform integrated studies using multiple markers in polychromatic flow cytometry assays.

**Figure 1:**
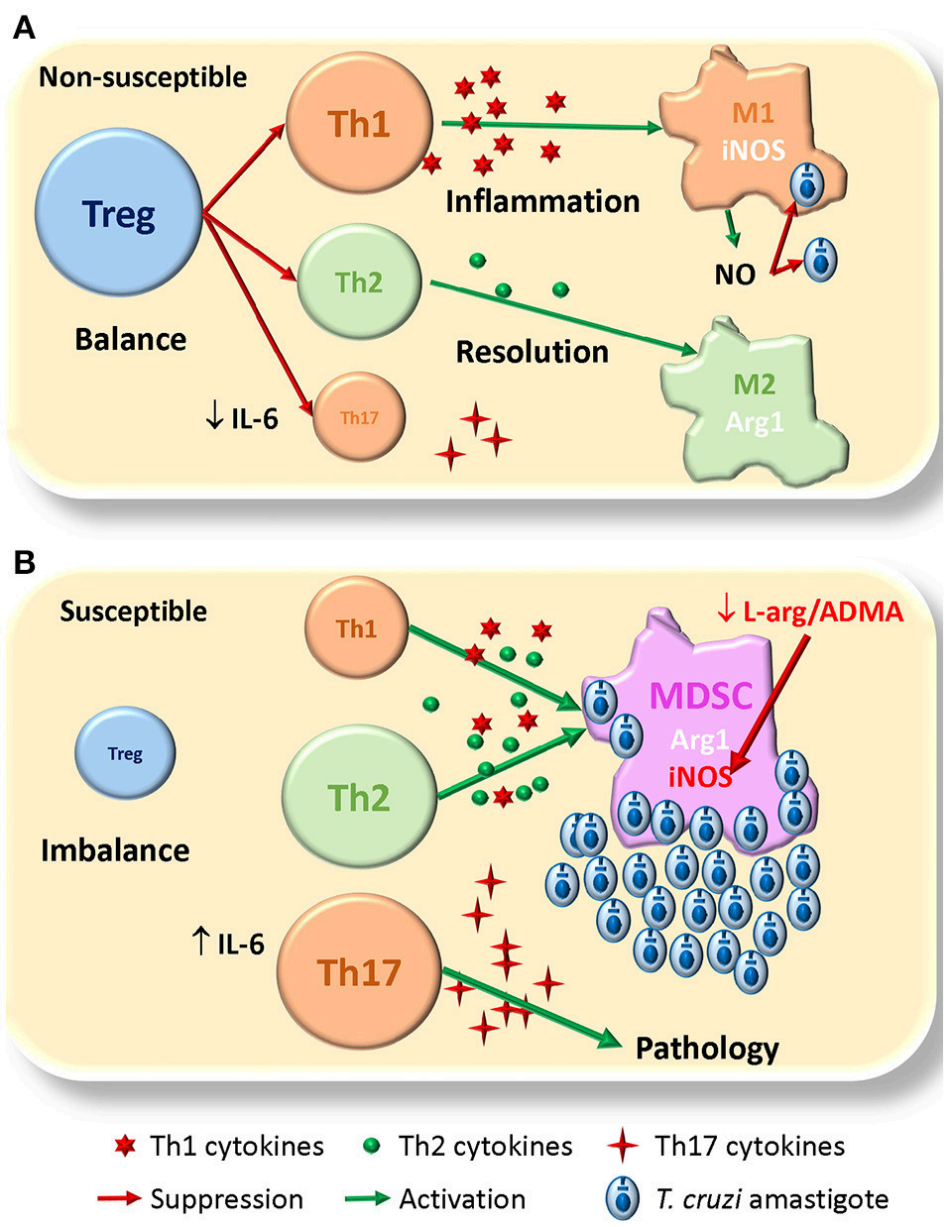
Proposed model for susceptibility to *T. cruzi* infection. **(A)** In the non-susceptible scenario, Treg cells allow a balanced Th1/Th2 immune response, able to control parasite replication by Th1/M1 response than later shifts to Th2/M2 for resolution of inflammation and healing damaged tissues; Th17 cells play a protective role. **(B)** In the susceptible context the lack of Treg cells conduces to an unbalanced immune response were MDSCs expand and consume L-arginine pools, that together with high levels of ADMA, inhibit NO production by iNOS losing the control of parasite replication; there is also expansion of pathogenic Th17. The size of the different cells (colored circles with names) denotes the magnitude of their expansion. Green arrows indicate activation. Red arrows indicate suppression/inhibition.

Thus, based in our findings and others, we propose a dual model for non-susceptibility/susceptibility to infection. In the non-susceptible model (Figure 1A) the expansion of Treg cells would balance the immune response allowing first inflammation mediated by Th1 and M1 cells that control parasite replication mediated by iNOS mediated NO production; this is followed by resolution of inflammation and healing of damaged tissues mediated by the Th2 and M2 cells, with some protective effect of Th17 cells. In the susceptible model (Figure 1B), the lack of Treg cells will imbalance the immune response, with exacerbation of Th1 and Th2 responses that interfere with macrophage activation, and give rise to the expansion of MDSCs; these cells deplete L-arginine pools, which together with ADMA high levels, inhibit NO production by iNOS and increases parasite replication; in this scenario, Th17 might expand and become pathogenic.

Thus, we have only little pieces of the puzzle since studies that integrate all the immune cells involved have not been performed. Finally, further studies combining “OMIC” techniques including genomics, transcriptomics, proteomics, and metabolomics studies, should shed more light in the role of immune regulation during *T. cruzi* infection.

## Author contributions

NG and MF wrote the revision and critically revised the manuscript. NG designed the figures and tables.

### Conflict of interest statement

The authors declare that the research was conducted in the absence of any commercial or financial relationships that could be construed as a potential conflict of interest.
